# Class II malocclusion treatment changes with the Jones jig, Distal jet and First Class appliances

**DOI:** 10.1590/1678-7757-2019-0364

**Published:** 2020-04-27

**Authors:** Lorena VILANOVA, José Fernando Castanha HENRIQUES, Mayara Paim PATEL, Rachelle Simões REIS, Roberto Henrique da Costa GREC, Aron ALIAGA-DEL CASTILLO, Silvio Augusto BELLINI-PEREIRA, Guilherme JANSON

**Affiliations:** 1 Universidade de São Paulo Faculdade de Odontologia de Bauru Departamento de Odontopediatria, Ortodontia e Saúde Coletiva BauruSão Paulo Brasil Universidade de São Paulo , Faculdade de Odontologia de Bauru , Departamento de Odontopediatria, Ortodontia e Saúde Coletiva , Bauru , São Paulo , Brasil .

**Keywords:** Cephalometry, Angle Class II malocclusion, Corrective orthodontics, Orthodontic appliances

## Abstract

**Objective:**

Maxillary molar distalization with intraoral distalizer appliances is a non-extraction orthodontic treatment used to correct molar relationship in patients with Class II malocclusion presenting maxillary dentoalveolar protrusion and minor skeletal discrepancies. This study compares the changes caused by three distalizers with different force systems.

**Methodology:**

71 patients, divided into three groups, were included. The Jones jig group (JJG, n=30; 16 male, 14 female, 13.17 years mean age) was treated with the Jones jig for 0.8 years. The Distal jet group (DJG, n=25; 8 male, 17 female, 12.57 years mean age) was treated with the Distal jet for 1.06 years. The First Class group (FCG, n=16; 6 male, 10 female, 12.84 years mean age) was treated with the First Class for 0.69 years. Intergroup treatment changes were compared using one-way ANOVA, followed by post-hoc Tukey’s tests.

**Results:**

Intergroup comparisons showed significantly greater maxillary incisor protrusion in DJG than in FCG (2.56±2.24 mm vs. 0.74±1.39mm, p=0.015). The maxillary first premolars showed progressive and significantly smaller mesial angulation in JJG, FCG and DJG, respectively (14.65±6.31º, 8.43±3.99º, 0.97±3.16º; p<0.001). They also showed greater mesialization in JJG than FCG (3.76±1.46 mm vs. 2.27±1.47 mm, p=0.010), and greater extrusion in DJG compared to JJG (0.90±0.77 mm vs 0.11±0.60 mm, p=0.004). The maxillary second premolars showed progressive and significantly smaller mesial angulation and mesialization in JJG, FCG and DJG, respectively (12.77±5.78º, 3.20±3.94º, -2.12±3.71º and 3.87±1.34 mm, 2.25±1.40 mm, 1.24±1.26 mm, respectively; p<0.001). DJG showed smaller distal angulation of maxillary first molars (-2.14±5.09º vs. -7.73±4.28º and -6.05±3.76º, for the JJG and FCG, respectively; p<0.001) and greater maxillary second molars extrusion (1.17±1.41 mm vs -0.02±1.16 mm and 0.16±1.40 mm, for the JJG and FCG, respectively; p=0.003). Overjet change was significantly larger in DJG compared to FCG (1.79±1.67 mm vs 0.68±0.84; p=0.046). Treatment time was smaller in FCG (0.69±0.22 years vs 0.81±0.33 years and 1.06±0.42 years, comparing it with the JJG and DJG, respectively; p=0.005).

**Conclusion:**

The three appliances corrected the Class II molar relationship by dentoalveolar changes. The Distal jet produced smaller molar distal angulation than the Jones jig and First Class. The First Class appliance showed less anchorage loss, greater percentage of distalization and shorter treatment time than the Jones jig and Distal jet.

## Introduction

Distalization of maxillary molars is indicated to treat Class II malocclusion without extractions in patients with maxillary dentoalveolar discrepancy and minor skeletal discrepancies. ^1^ Headgear ^2^ and Wilson maxillary bimetric distalizing arch system ^3^ have been widely used in the past, however these distalizing appliances require the patient’s compliance to achieve molar distal movement. Protocols that require less patient cooperation are more effective and predictable. ^4^

Several fixed and intraoral appliances for maxillary molars distalization have been described as an option to reduce the need of patient compliance. Most of these appliances involve an anchorage unit, commonly an acrylic Nance button, and an active unit. The active components can be repelling magnets, ^5^ superelastic nickel-titanium (NiTi) archwires, ^6^ coil springs on continuous archwire or on sectional archwire, ^7 , 8^ springs in beta titanium alloy, ^9^ and vestibular screws associated with palatal NiTi coil springs. ^10^

These intraoral distalizers are practical resources to correct Class II molar relationship in a shorter time. ^8 , 11^ The amount of maxillary molar movement and subsequent side effects could be directly associated with the biomechanics and particularities of each appliance. The Jones jig is a buccal distalization appliance whereas the Distal jet applies a palatal distalization force. Some advantages of the Distal jet have been reported such as the ability to promote molar distalization with less angulation effects, because the distalizing force applied is closer to the molar center of resistance. ^8^ More recently, the First Class was proposed as an intraoral appliance with a palatal and buccal force system. ^10^

The dentoalveolar and skeletal changes of these appliances have been previously investigated. ^4 , 7 , 8 , 10 , 12 , 13^ However, no previous studies directly compared the changes among treatments. Therefore, this study cephalometrically compares the dentoalveolar, skeletal and soft tissue effects of three appliances with different force systems (Jones jig, Distal jet and First Class) used for maxillary molar distalization in Class II malocclusion patients.

## Methodology

This retrospective study was approved by the Research Ethics Committee of Bauru School of Dentistry, University of São Paulo. Informed consent was signed by all patients’ parents or legal guardians allowing their treatment and participation in the study.

Sample size was calculated considering a mean difference of 1.6 mm between groups for the amount of distal movement of maxillary molars in the sagittal plane, contemplated as the primary outcome, with a previously reported standard deviation of 1.5 mm, ^10^ using 80% test power, at 5% alpha level. Then, a minimum of 16 patients was necessary in each group.

The selection criteria included patients with at least ¼ cusp Class II molar relationship, ^14^ all permanent teeth up to the first molars erupted, no severe mandibular crowding, no crossbite, no anterior open bite, no agenesis, supernumerary or tooth loss and no previous orthodontic intervention. Each group was treated in different periods. Patients were allocated to each group when they satisfied the selection criteria. The sample consisted of 71 patients divided into 3 groups. All groups were treated with distalization appliances using conventional anchorage. Most of the patients had erupted maxillary second molars.

**Figure 1:**
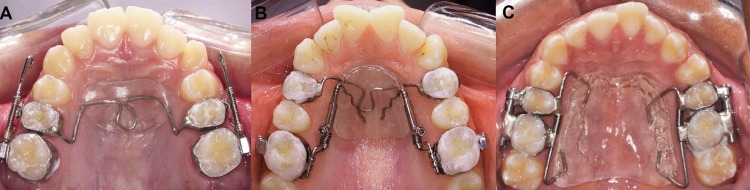
Distalization appliances. A: Jones jig; B: Distal jet; C: First Class

The Jones jig group (JJG) consisted of 30 patients (16 male, 14 female) with 13.17±1.24 years initial mean age. The NiTi coil spring (G&H Wire Co, Greenwood, Indiana, USA) was activated 5 mm every 4 weeks to deliver 125 g of force. A Nance button, cemented on the second premolars, was used as anchorage ( Figure 1A ).

The Distal jet group (DJG) consisted of 25 patients (8 male, 17 female) with 12.57±1.43 years initial mean age. In this appliance, the Nance button was cemented on the maxillary first premolars serving as anchorage ( Figure 1B ). Different amounts of force (240g or 180g) were applied based on the clinical presence or absence of the second molars. The greatest force was used when second molars were erupted. ^4^ The device was reactivated once a month in the same manner.

The First Class group (FCG) consisted of 16 subjects (6 male, 10 female) with 12.84±1.31 years initial mean age. The First Class appliance consisted of two buccal-activation screws (10 mm long) soldered to the first molar bands and placed in closed rings soldered to the second premolar bands, two 0.010x0.045-inch palatal open NiTi coil springs (10 mm long) and a modified Nance button ( Figure 1C ). The buccal screws were activated a quarter turn in a counterclockwise direction once a day, activating 0.1 mm *per* day. ^10^

Three orthodontic graduate students, supervised by the same professor, performed the treatment of all patients. Each group was treated by only one operator. In all groups, distalization was performed until a super-Class I molar relationship was obtained. ^5^

**Figure 2:**
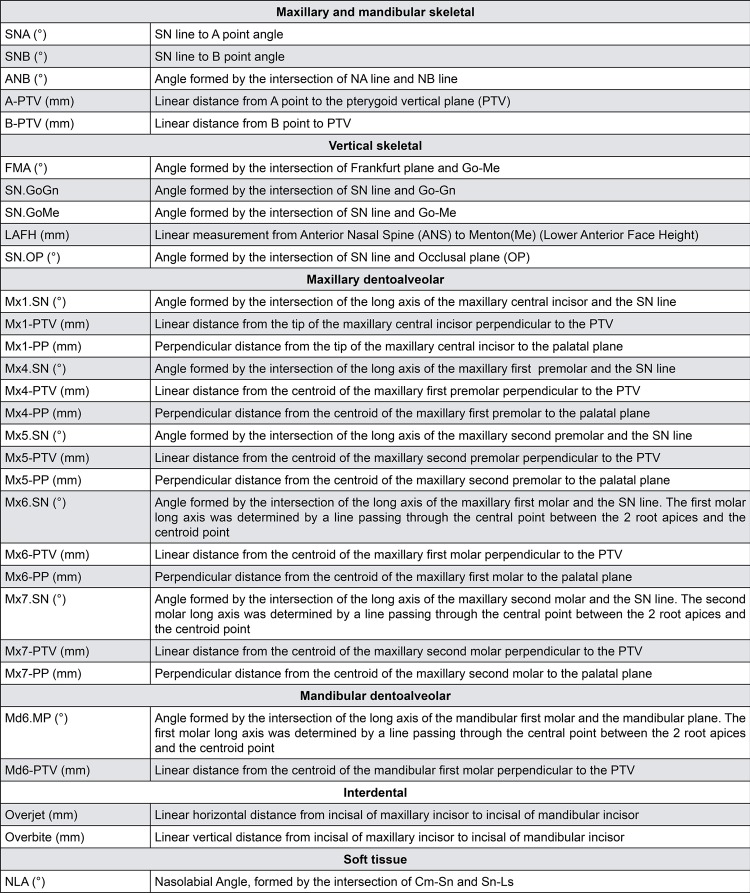
Cephalometric measurements

**Figure 3:**
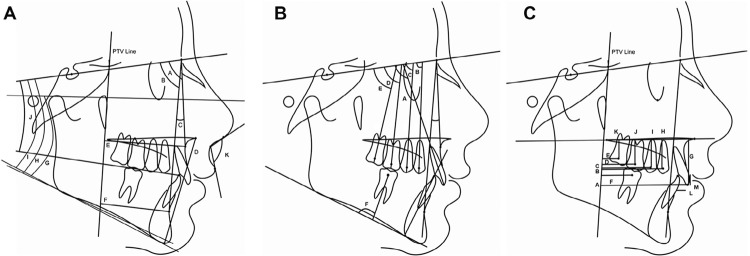
Cephalometric variables. A: Skeletal and soft tissue variables (A. SNA; B.SNB; C. ANB; D. ANS-Me; E. A-PTV; F. B-PTV; G. FMA; H. SN.GoGn; I. SN.GoMe; J. SN.Occlusal plane; K. Nasolabial angle); B: Angular dental variables (A. Mx1.SN; B. Mx4.SN; C. Mx5.SN; D. Mx6.SN; E. Mx7.SN; F. Md6.MP); C: Linear dental variables (A. Mx1-PTV; B. Mx4-PTV; C. Mx5-PTV; D. Mx6-PTV; E. Mx7-PTV; F. Md6-PTV; G. Mx1-PP; H. Mx4-PP; I. Mx5-PP; J. Mx6-PP; K. Mx7-PP; L. Overjet; M. Overbite)

Lateral head films were obtained at pretreatment (T1) and after molar distalization (T2). They were analyzed with Dentofacial Planner 7.02 software (Dentofacial Planner, Toronto, Ontario, Canada). The image magnification factors were corrected by the software. A total of 30 variables were evaluated on each cephalogram ( Figures 2 and 3 ). Bilateral structures of interest were averaged.

### Error study

In total, 42 cephalograms were randomly selected and retraced by the same examiner (L.V.) after a 1-month interval. The random errors were evaluated using Dahlberg’s formula ( *S* ^2^ *= Σd* ^2^ */2n* ), where *S* ^2^ is the error variance and *d* is the difference between two determinations of the same variable. The systematic errors were assessed with dependent t-tests at p<0.05. The random errors ranged between 0.50 mm (Mx1-PP) and 1.18 mm (LAFH) and between 0.52 (ANB) and 2.80 (NLA) degrees that were within acceptable limits, ^13^ and only one variable (A-PTV) demonstrated a significant systematic error.

### Statistical analyses

Normal distributions were confirmed with Kolmogorov-Smirnov tests. Intergroup comparability regarding sex distribution, severity of Class II malocclusion and the number of erupted maxillary second molars were assessed with Chi-square tests.

Initial and final ages, treatment time, cephalometric statuses at pretreatment and treatment changes were compared between groups using one-way Analysis of Variance (ANOVA), followed by Tukey’s tests.

Statistica software (Statistica for Windows, version 6.0, Statsoft, Tulsa, Oklahoma, USA) was used to perform all statistical analyses. Statistical significance was set at p<0.05.

Considering the anchorage loss of premolars and incisors, the effect of molar distalization in the total movement in the sagittal dimension, as reported by Kinzinger, et al. ^15^ (2008), were also calculated as percentages.

## Results

The groups were comparable regarding sex and Class II malocclusion severity distributions, number of erupted maxillary second molars, initial and final mean ages ( Table 1 ). However, the First Class group presented a shorter treatment time than the Distal jet group.

**Table 1:** Comparison of sex and Class II malocclusion severity distributions, amount of erupted maxillary second molars, initial and final ages and treatment times

Variable	JJ-Jones jig n=30		DJ-Distal jet n=25		FC-First Class n=16		P
Sex							
Male	16 (53.3%)		8 (32%)		6 (37.5%)		0.254 ^€^
Female	14 (46.7%)		17 (68%)		10 (62.5%)		
Occlusal malocclusion severity							
¼ cusp Class II	7 (23%)		6 (24%)		6 (37.5%)		0.414 ^€^
½ cusp Class II	14 (47%)		16 (64%)		8 (50%)		
¾ cusp Class II	5 (17%)		3 (12%)		1 (6.25%)		
Full cusp Class II	4 (13%)		0 (0%)		1 (6.25%)		
Erupted second molars							
Erupted	24 (80%)		17 (68%)		12 (75%)		0.596 ^€^
Unerupted	6 (20%)		8 (32%)		4 (25%)		

	**Mean**	**SD**	**Mean**	**SD**	**Mean**	**SD**	

Initial age	13.17 ^A^	1.24	12.57 ^A^	1.29	12.84 ^A^	1.31	0.254 ^¥^
Final age	14.04 ^A^	1.29	13.64 ^A^	1.60	13.53 ^A^	1.38	0.421 ^¥^
Treatment time	0.81 ^AB^	0.33	1.06 ^A^	0.42	0.69 ^B^	0.22	0.005 ^¥*^

€Chi-Square test; ¥ANOVA

*Statistically significant at P<0.05

Mean values and standard deviations of all variables at pre-treatment (T1) and posttreatment (T2) are shown in Table 2 .

**Table 2:** Mean values and standard deviations (SD) of all variables at pretreatment (T1) and posttreatment (T2)

	JJ (Jones jig) Group (n=30)				DJ (Distal jet) Group (n=25)				FC (First Class) Group (n=16)			
Variables	T1	SD	T2	SD	T1	SD	T2	SD	T1	SD	T2	SD
	Means		Means		Means		Means		Means		Means	
**Maxillary and mandibular skeletal**												

SNA	83.97	3.32	84.19	3.14	82.22	5.28	82.67	5.60	85.39	4.20	85.45	4.11
SNB	80.20	3.12	80.41	3.06	79.02	3.90	79.18	4.19	79.05	3.85	79.21	4.48
ANB	3.77	2.30	3.77	1.94	4.48	2.87	4.77	2.68	6.34	2.05	6.25	1.79
A-PTV	48.10	3.48	48.31	3.36	48.13	2.49	48.39	2.76	50.90	3.18	50.80	3.13
B-PTV	46.54	5.11	46.72	5.11	46.92	3.31	47.06	3.62	47.76	5.99	48.35	6.36

**Vertical skeletal**												

FMA	26.74	5.00	26.83	5.06	26.83	3.64	27.20	4.36	27.35	5.09	27.74	5.66
SN.GoGn	30.31	4.30	30.59	4.24	30.35	3.85	30.69	4.53	30.98	4.49	30.47	5.26
SN.GoMe	26.05	5.71	26.45	5.96	25.69	4.41	25.92	5.27	25.87	5.41	26.68	6.11
LAFH	61.81	5.12	63.48	5.71	61.43	5.09	63.88	6.52	63.64	6.23	65.04	6.37
SN.OP	9.93	4.64	10.59	4.58	11.27	3.71	12.00	4.35	11.35	4.05	11.25	4.16

**Maxillary dentoalveolar**												

Mx1.SN	109.60	5.08	115.68	5.14	107.30	6.41	112.62	7.54	110.11	7.49	115.21	6.8
Mx1-PTV	55.32	4.81	57.41	4.92	55.81	3.57	58.37	4.71	59.03	4.33	59.77	4.11
Mx1-PP	27.00	2.40	26.89	2.76	27.08	2.75	27.44	3.05	27.08	2.53	27.38	2.49
Mx4.SN	88.84	4.99	103.49	4.77	85.66	5.19	86.63	4.94	84.35	6.12	92.78	7.54
Mx4-PTV	36.32	3.69	40.08	3.94	36.76	2.68	40.13	2.87	38.83	3.99	41.10	4.57
Mx4-PP	19.87	2.20	19.98	2.39	20.29	2.25	21.19	2.31	20.16	2.41	20.72	2.33
Mx5.SN	80.41	4.85	93.18	5.52	79.16	4.80	77.04	5.66	77.76	5.64	80.96	7.83
Mx5-PTV	29.70	3.48	33.57	3.72	29.82	2.64	31.06	2.75	31.90	4.03	34.15	4.45
Mx5-PP	19.24	2.04	19.72	2.25	19.50	2.12	19.68	2.26	19.34	2.58	20.14	2.27
Mx6.SN	71.89	5.33	64.16	5.45	70.97	5.23	68.83	5.57	70.83	4.50	64.78	5.99
Mx6-PTV	21.32	3.47	19.50	3.47	21.37	2.80	19.85	2.78	23.58	3.90	21.10	3.60
Mx6-PP	17.29	2.36	16.68	2.35	17.79	2.24	17.98	2.70	18.13	2.32	17.91	2.31
Mx7.SN	62.82	6.52	56.15	7.13	63.16	4.94	56.97	5.19	63.93	5.59	57.66	6.66
Mx7-PTV	11.99	3.04	10.59	3.25	12.19	2.40	10.24	2.50	13.87	3.36	11.78	3.60
Mx7-PP	12.50	3.66	12.48	3.35	12.88	3.55	14.05	3.28	13.53	3.41	13.69	3.28

**Mandibular dentoalveolar**												

Md6.MP	78.94	4.28	78.49	7.77	78.60	4.11	79.00	4.12	78.93	4.47	76.30	14.59
Md6-PTV	21.32	3.47	21.69	3.69	21.37	2.80	21.96	2.68	23.58	3.90	23.83	3.97

**Interdental**												

Overjet	4.84	1.66	6.23	2.03	5.25	1.57	7.04	2.26	6.12	2.47	6.80	2.88
Overbite	3.78	1.58	2.95	1.76	3.58	1.83	2.78	2.10	3.71	1.83	2.86	2.42

**Soft tissue**												

NLA	103.06	11.30	99.62	10.50	99.56	14.69	99.18	14.28	101.24	7.50	99.16	8.93

At pretreatment, the First Class group had significantly greater skeletal Class II relationship, maxillary length, and maxillary incisors protrusion than the other groups ( Table 3 ). The first premolar mesial angulation was progressive and significantly smaller in the Jones jig, Distal jet and First Class groups, respectively.

**Table 3:** Pretreatment intergroup cephalometric comparison (ANOVA followed by Tukey’s tests)

Variables	JJ (Jones jig) Group (n=30)		DJ (Distal jet) Group (n=25)		FC (First Class) Group (n=16)		P
	Mean	SD	Mean	SD	Mean	SD	
**Maxillary and mandibular skeletal**							

SNA	83.97 ^A^	3.32	82.22 ^A^	5.28	85.39 ^A^	4.20	0.292
SNB	80.20 ^A^	3.12	79.02 ^A^	3.90	79.05 ^A^	3.85	0.401
ANB	3.77 ^A^	2.30	4.48 ^A^	2.87	6.34 ^B^	2.05	0.004*
A-PTV	48.10 ^A^	3.48	48.13 ^A^	2.49	50.90 ^B^	3.18	0.009*
B-PTV	46.54 ^A^	5.11	46.92 ^AB^	3.31	47.76 ^B^	5.99	0.712

**Vertical skeletal**							

FMA	26.74 ^A^	5.00	26.83 ^A^	3.64	27.35 ^A^	5.09	0.908
SN.GoGn	30.31 ^A^	4.30	30.35 ^A^	3.85	30.98 ^A^	4.49	0.858
SN.GoMe	26.05 ^A^	5.71	25.69 ^A^	4.41	25.87 ^A^	5.41	0.967
LAFH	61.81 ^A^	5.12	61.43 ^A^	5.09	63.64 ^A^	6.23	0.414
SN.OP	9.93 ^A^	4.64	11.27 ^A^	3.71	11.35 ^A^	4.05	0.402

**Maxillary dentoalveolar**							

Mx1.SN	109.60 ^A^	5.08	107.30 ^A^	6.41	110.11 ^A^	7.49	0.266
Mx1-PTV	55.32 ^A^	4.81	55.81 ^A^	3.57	59.03 ^B^	4.33	0.020*
Mx1-PP	27.00 ^A^	2.40	27.08 ^A^	2.75	27.08 ^A^	2.53	0.991
Mx4.SN	88.84 ^A^	4.99	85.66 ^B^	5.19	84.35 ^c^	6.12	0.015*
Mx4-PTV	36.32 ^A^	3.69	36.76 ^A^	2.68	38.83 ^A^	3.99	0.062
Mx4-PP	19.87 ^A^	2.20	20.29 ^A^	2.25	20.16 ^A^	2.41	0.777
Mx5.SN	80.41 ^A^	4.85	79.16 ^A^	4.80	77.76 ^A^	5.64	0.234
Mx5-PTV	29.70 ^A^	3.48	29.82 ^A^	2.64	31.90 ^A^	4.03	0.086
Mx5-PP	19.24 ^A^	2.04	19.50 ^A^	2.12	19.34 ^A^	2.58	0.913
Mx6.SN	71.89 ^A^	5.33	70.97 ^A^	5.23	70.83 ^A^	4.50	0.728
Mx6-PTV	21.32 ^A^	3.47	21.37 ^A^	2.80	23.58 ^A^	3.90	0.071
Mx6-PP	17.29 ^A^	2.36	17.79 ^A^	2.24	18.13 ^A^	2.32	0.469
Mx7.SN	62.82 ^A^	6.52	63.16 ^A^	4.94	63.93 ^A^	5.59	0.825
Mx7-PTV	11.99 ^A^	3.04	12.19 ^A^	2.40	13.87 ^A^	3.36	0.100
Mx7-PP	12.50 ^A^	3.66	12.88 ^A^	3.55	13.53 ^A^	3.41	0.646

**Mandibular dentoalveolar**							

Md6.MP	78.94 ^A^	4.28	78.60 ^A^	4.11	78.93 ^A^	4.47	0.951
Md6-PTV	21.32 ^A^	3.47	21.37 ^A^	2.80	23.58 ^A^	3.90	0.128

**Interdental**							

Overjet	4.84 ^A^	1.66	5.25 ^A^	1.57	6.12 ^A^	2.47	0.088
Overbite	3.78 ^A^	1.58	3.58 ^A^	1.83	3.71 ^A^	1.83	0.906

**Soft tissue**							

NLA	103.06 ^A^	11.30	99.56 ^A^	14.69	101.24 ^A^	7.50	0.559

*Statistically significant at P<0.05

During treatment, the maxillary incisors showed significantly greater protrusion in the Distal jet than in the First Class group ( Table 4 ).

**Table 4:** Intergroup treatment changes comparison (ANOVA followed by Tukey tests)

Variables	JJ (Jones jig) Group (n=30)		DJ (Distal jet) Group (n=25)		FC (First Class) Group (n=16)		P
	Mean	SD	Mean	SD	Mean	SD	
**Maxillary and mandibular skeletal**							

SNA	0.22 ^A^	0.96	0.45 ^A^	1.20	0.06 ^A^	1.11	0.516
SNB	0.21 ^A^	0.70	0.16 ^A^	1.39	0.16 ^A^	1.04	0.978
ANB	0.00 ^A^	0.90	0.29 ^A^	0.66	-0.09 ^A^	0.89	0.278
A-PTV	0.21 ^A^	0.62	0.26 ^A^	0.68	-0.10 ^A^	0.76	0.208
B-PTV	0.18 ^A^	0.89	0.14 ^A^	1.05	0.59 ^A^	2.08	0.512

**Vertical skeletal**							

FMA	0.09 ^A^	1.13	0.37 ^A^	2.03	0.39 ^A^	1.86	0.774
SN.GoGn	0.28 ^A^	1.86	0.34 ^A^	1.45	-0.51 ^A^	1.34	0.201
SN.GoMe	0.40 ^A^	1.91	0.23 ^A^	2.02	0.81 ^A^	2.23	0.668
LAFH	1.67 ^A^	1.17	2.45 ^A^	2.23	1.40 ^A^	1.28	0.094
SN.OP	0.66 ^A^	2.31	0.73 ^A^	2.11	-0.10 ^A^	1.37	0.402

**Maxillary dentoalveolar**							

Mx1.SN	6.08 ^A^	3.86	5.32 ^A^	4.24	5.10 ^A^	2.63	0.640
Mx1-PTV	2.09 ^AB^	1.88	2.56 ^A^	2.24	0.74 ^B^	1.39	0.015*
Mx1-PP	-0.11 ^A^	1.11	0.36 ^A^	1.08	0.30 ^A^	0.96	0.210
Mx4.SN	14.65 ^A^	6.31	0.97 ^B^	3.16	8.43 ^C^	3.99	<0.001*
Mx4-PTV	3.76 ^A^	1.46	3.37 ^AB^	1.67	2.27 ^B^	1.47	0.010*
Mx4-PP	0.11 ^A^	0.60	0.90 ^B^	0.77	0.56 ^AB^	1.32	0.004*
Mx5.SN	12.77 ^A^	5.78	-2.12 ^B^	3.71	3.20 ^c^	3.94	<0.001*
Mx5-PTV	3.87 ^A^	1.34	1.24 ^B^	1.26	2.25 ^C^	1.40	<0.001*
Mx5-PP	0.48 ^A^	0.81	0.18 ^A^	0.76	0.80 ^A^	1.57	0.161
Mx6.SN	-7.73 ^A^	4.28	-2.14 ^B^	5.09	-6.05 ^A^	3.76	<0.001*
Mx6-PTV	-1.82 ^A^	1.33	-1.52 ^A^	1.51	-2.48 ^A^	0.93	0.080
Mx6-PP	-0.61 ^A^	0.97	0.19 ^A^	1.35	-0.22 ^A^	1.47	0.061
Mx7.SN	-6.67 ^A^	6.09	-6.19 ^A^	5.04	-6.27 ^A^	4.39	0.940
Mx7-PTV	-1.40 ^A^	1.41	-1.95 ^A^	1.33	-2.09 ^A^	1.43	0.190
Mx7-PP	-0.02 ^A^	1.16	1.17 ^B^	1.41	0.16 ^A^	1.40	0.003*

**Mandibular dentoalveolar**							

Md6.MP	-0.45 ^A^	2.35	0.40 ^A^	3.33	-2.63 ^A^	13.27	0.367
Md6-PTV	0.37 ^A^	0.63	0.59 ^A^	0.66	0.25 ^A^	1.16	0.366

**Interdental**							

Overjet	1.39 ^AB^	1.28	1.79 ^A^	1.67	0.68 ^B^	0.84	0.046*
Overbite	-0.83 ^A^	1.01	-0.80 ^A^	1.04	-0.85 ^A^	1.14	0.989

**Soft tissue**							

NLA	-3.44 ^A^	5.42	-0.38 ^A^	5.41	-2.08 ^A^	5.76	0.130

*Statistically significant at P<0.05

The maxillary first premolars showed progressive and significantly smaller mesial angulation in the Jones jig, First Class and Distal jet groups, respectively. They also showed significantly greater mesialization in the Jones jig than in the First Class group, and significantly greater extrusion in the Distal jet than in the Jones jig group ( Table 4 ).

The maxillary second premolars showed progressive and significantly smaller mesial angulation and mesialization in the Jones jig, First Class and Distal jet groups, respectively ( Table 4 ).

The maxillary first molar distal angulation was significantly smaller in the Distal jet than in the other groups. The extrusion of maxillary second molars was significantly greater in the Distal jet than in the other groups ( Table 3 ).

The overjet change was significantly larger in the Distal jet than in the First Class group ( Table 4 ).

The First Class group showed greater percentages of maxillary molar distalization considering the anchorage loss of premolars and incisors, followed by the Jones jig and the Distal jet ( Tables 5 and 6 ).

**Table 5:** Percentages of molar distalization in the total movement in the sagittal dimension and anchorage loss considering premolars

	DISTALIZATION		ANCHORAGE LOSS			
**APPLIANCE**	Distal movement of maxillary first molars Variable: Mx6-PTV		Mesial movement of maxillary premolars Maxillary second premolars (Jones Jig and First Class) Variable: Mx5-PTV Maxillary first premolars (Distal Jet) Variable: Mx4-PTV		TOTAL AMOUNT	

	**mm**	**%**	**mm**	**%**	**mm**	**%**

JJ (Jones Jig) Group	1.82	31.99	3.87	68.01	5.69	100
DJ (Distal Jet) Group	1.52	31.08	3.37	68.92	4.89	100
FC (First Class) Group	2.48	52.43	2.25	47.57	4.73	100

**Table 6:** Percentages of molar distalization in the total movement in the sagittal dimension and anchorage loss considering the incisors

	DISTALIZATION		ANCHORAGE LOSS			
**APPLIANCE**	Distal movement of maxillary first molars Variable: Mx6-PTV		Mesial movement of maxillary incisors Variable: Mx1-PTV		TOTAL AMOUNT	

	**mm**	**%**	**mm**	**%**	**mm**	**%**

JJ (Jones Jig) Group	1.82	46.55	2.09	53.45	3.91	100
DJ (Distal Jet) Group	1.52	37.25	2.56	62.75	4.08	100
FC (First Class) Group	2.48	77.02	0.74	22.98	3.22	100

## Discussion

Previous clinical studies and systematic reviews have investigated the changes resulting from intraoral molar distalizers. However, inter-study comparisons are limited because of their heterogeneity. ^15 - 17^ This study is relevant since it evaluates three distalizing appliances with different force systems to directly compare their treatment effects. The sample size on each group was similar to other previous studies. ^1 , 4 , 11 , 18 - 20^

Considering the number of variables used in this study, one could argue that Bonferroni corrections should be used. ^21^ Nevertheless, this would decrease the probability of detecting slight significant differences between groups, which are very important in these comparisons. Since the focus of this study was to investigate whether there is a minimum difference in the treatment changes between the three groups, Bonferroni corrections were not performed.

The groups were reasonably similar at T1 ( Table 3 ). The more accentuated Class II maxillomandibular relationship in the First Class Group was probably due to the greater maxillary length that this group presented. Consequently, the maxillary incisor also presented greater protrusion in this group. The mesial angulation of the maxillary first premolars was progressive and significantly smaller in the Jones jig, Distal jet and First Class groups, respectively ( Table 3 ). However, these characteristics do not interfere with the comparison of results of the treatment changes since they do not affect the appliance performance.

The shorter treatment time in the First Class group was similar to previously reported results. ^12^

Similar changes of the skeletal variables were observed between groups, as expected, because these treatment protocols do not promote significant changes on skeletal structures, as previously demonstrated ^13 , 22 , 23^ ( Table 4 ).

Commonly, the undesirable effects produced by these appliances include mesialization and mesial angulation of premolars and protrusion and labial inclination of the anterior teeth, as reported by Kinzinger, et al. ^15^ (2008), and Antonarakis and Kiliaridis ^16^ (2008). The Distal jet presented significantly greater maxillary incisor protrusion compared to the First Class ( Table 4 ). This difference could be explained by the greater anchorage unit used in the First Class group. Since the modified Nance button is attached to the maxillary first molars and second premolars, more teeth are included as anterior anchorage for molar distalization. Furthermore, the Nance button is also larger in this appliance. ^12^

Mesial angulation of maxillary first premolars was progressive and significantly smaller in the Jones jig, First Class and Distal jet groups, respectively. Significantly greater first premolars mesial angulation in the Jones jig group has been reported in previous studies as result of anchorage loss. ^1 , 13 , 17 , 22 , 24^ The maxillary first premolars showed significantly smaller mesial angulation in the Distal jet, despite these teeth served as the anchorage unit in this appliance. ^19^ However, as the premolar bands were attached to the Nance button, this prevented them from excessive mesial tipping. ^4 , 15^

The significantly greater mesialization of the maxillary first premolars in the Jones jig than in the First Class could also be explained by the larger Nance button in the First Class, representing a greater anchorage unit. ^12^ The First Class results are in accordance with a previous study. ^10^

The Distal jet presented greater extrusion of maxillary first premolars than the Jones jig. This is probably because the first premolars are attached to the appliance. As the resulting mesial force on these teeth finds resistance to mesial movement by the anterior teeth and their tipping is restricted, there is a resultant vertical vector which causes extrusion of these teeth. ^4^ Vertical movements of premolars could be expected. ^16^ However, they play a minor part and should not be considered clinically significant. ^15^

Mesial angulation of maxillary second premolars were progressively smaller in the Jones jig and First Class, as expected because of the smaller and larger anchorage units, respectively. ^1 , 13 , 17 , 22 , 24^ In contrast to the these groups, the Distal jet showed distal angulation of the maxillary second premolar. Evaluation of dental casts in a previous study demonstrated similar results. ^4^ Differently from the other two appliances, the second premolars are not attached to the appliance. Therefore, as the molars distalize, the second premolars are pulled by the transeptal fibers and experience some distal tipping. ^25^

The Jones Jig group presented significantly greater mesialization of the second premolars than the other groups. This could be explained by the smaller Nance button used in this group. Moreover, the smallest mesial movement of the second premolars in the Distal jet group was expected since these teeth were not incorporated in the anchorage unit in this appliance.

The Distal jet presented smaller distal angulation of maxillary first molars than the other groups. According to other studies, this could be explained by the appliance design. The force is applied on the palatal side, more cervically to the first molar crown, compared to the other appliances, producing forces parallel and closer to the center of resistance, resulting in greater bodily movement, ^4 , 8 , 15^ and smaller distal inclination, as mentioned by Antonarakis and Kiliaridis ^16^ (2008). Even with the small amount of distal movement of this group, distal angulation was observed. This reflects that Distal jet appliances might decrease the distal angulation effect, but it cannot neutralize the effect. ^15 , 16^

It seems that decreasing the forces for maxillary molar distalization has not been effective to reduce the molar distal angulation. In this study, the Jones jig appliance used with 125g force, demonstrated similar maxillary molar distal angulation when compared with the 200g force used in the First Class appliance, and greater distal angulation when compared with the 180 or 240g force used with the Distal jet. Previous studies evaluating the Jones jig appliance exerting 75g of distal force demonstrated similar results. ^1 , 11 , 22^

According to some authors, distal angulation of maxillary molars produces molar intrusion. ^18 , 26^ This could explain the greater, but not statistically significant, intrusive changes observed in the maxillary first molars with the Jones jig, since it presented greater amount of distal angulation. On the other hand, the Distal jet presented greater vertical development of the maxillary second molars in comparison to the other groups, similar to a previous study. ^4^ This could be explained by the greater treatment time of this group, which probably resulted in greater amount of eruption of maxillary second molars at the end of the distalization phase.

The overjet increased significantly in the Distal jet than in the First Class. This probably occurs for the first premolars are included in the anchorage unit in the Distal jet and but not included in the First Class appliance. As mentioned, this increased overjet caused, as a consequence, the greatest and smallest incisor protrusions, in these appliances, respectively.

Since changes in maxillary incisor angulation were similar in all groups and only the maxillary incisor protrusion was significantly greater in the Distal jet compared to the First Class, the lack of statistically significant difference between groups regarding the nasolabial angle could be expected, as previously reported ^27^ ( Table 4 ).

The greater percentage of molar distal movement, considering the anchorage loss, observed in the First Class group ( Tables 5 and 6 ) could be expected since this group presented numerically but not statistically significant greater amount of maxillary molar distalization, in mm, than the other groups ( Table 4 ). Similar results were observed in previous studies. ^10 , 12 , 15^

When analyzing the percentages of distal movement between the Jones jig and the Distal jet, both had similar percentages of molar distal movement, as expected, because the amount of distalization were very close. This was also reported by Antonarakis and Kiliaridis ^16^ (2008) who compared buccal and palatal distalization appliances. Despite the similarity between buccal and palatal appliances, it is important to mention that the Distal jet presented smaller distal inclination of maxillary molars, as reported by Antonarakis and Kiliaridis ^16^ (2008), as well.

Independently of the amount of maxillary molar distalization and anchorage loss, Class II molar relationship correction was observed in all patients after distalization mechanics with the three appliances.

The results indicate that the type of anchorage used in the studied appliances is insufficient to counteract the distalization forces. ^16 , 28 , 29^ Side effects should be expected during maxillary molar distalization with conventional anchorage either in the distalized molar or in the anchorage unit. ^15 , 16^ Recently, alternative anchorage designs using devices with skeletal anchorage have been described as reducing the side effects of distalization, thus they seem to be efficient alternatives for maxillary molar distalization. ^17 , 29 - 31^

Nonetheless, it is important to know the effects of the several distalization systems with and without skeletal anchorage to choose the ideal alternative, depending on the singular requirements of the patient.

After distalization, orthodontic mechanics must be complemented with fixed appliances to preserve the results of distalization and to correct its side effects. In general, maxillary molar distalization can be achieved with the three studied appliances. The device selection should depend on predictability, minimal undesirable side effects, cost-efficiency, and patient need.

Further studies with greater sample sizes should be performed to confirm our results. Moreover, long-term studies should be performed to evaluate treatment stability of these types of appliances. ^16^

## Conclusions

The three appliances efficiently corrected the Class II molar relationship by dentoalveolar changes with some undesirable effects;

The Distal jet presented significantly smaller molar distal angulation and smaller, but not statistically significant, amount of distalization than the Jones jig and First Class appliances;

The First Class appliance produced less anchorage loss, greater percentage of distalization, and shorter treatment time than the Jones jig and Distal jet appliances.
